# Let’s groove: attachment techniques of Eurasian elk (*Alces alces*) tooth pendants at the Late Mesolithic cemetery Yuzhniy Oleniy Ostrov (Lake Onega, Russia)

**DOI:** 10.1007/s12520-020-01237-5

**Published:** 2020-12-15

**Authors:** Kristiina Mannermaa, Riitta Rainio, Evgeny Yu. Girya, Dmitry V. Gerasimov

**Affiliations:** 1 Department of Cultures, Archaeology, University of Helsinki, P.O. Box 59, 00014 Helsinki, Finland; 2 Institute of History and Archaeology, University of Tartu, Jakobi 2, -206 Tartu, Estonia; 3 Russian Academy of Sciences, Institute for History of Material Culture, Saint Petersburg, Russia; 4 Russian Academy of Sciences, Peter the Great Museum of Anthropology and Ethnography, Saint Petersburg, Russia

**Keywords:** Mesolithic, Elk tooth pendants, Ornamentation, Kinships, Social values

## Abstract

More than 4300 Eurasian elk (*Alces alces*) incisors, most of them pendants, were found in 84 burials in the Late Mesolithic cemetery of Yuzhniy Oleniy Ostrov, Northwest Russia. We analysed the manufacture techniques of elk teeth (4014), in the collection of the Peter the Great Museum of Anthropology and Ethnography, St Petersburg. A striking observation is that the manufacture of these pendants is similar in all burials. Teeth were worked by carving one or several grooves around the root tip. In addition to grooved ones, a number of teeth were not worked at all. The uniformity of the chosen species, tooth and techniques indicates that strict norms prevailed in the pendant industry. Despite the overall similarity, our study shows some variation in making pendants. A groove can cut the whole circumference of the root, or several distinct grooves can mark opposite sides of the root. Sometimes the grooves are deep and made carefully, and sometimes they are weak and made hastily. A typology of various groove types was created. In many graves, one groove type dominates. We interpret that this inter-burial variation and domination of one type resulted from personal choice and taste based on practicality. Such variation could also be associated with kin identifiers, but we did not find clear support for that in our study. Our study indicates that the groove types as such had no connection with particular ornaments, garments or hanging positions.

## Introduction

### Tooth pendants and social structures

Cemeteries with rich burial finds and well-preserved human remains can be used to study the social structures of prehistoric hunter-gatherer societies. Such burial sites have been found in Scandinavia (e.g. Skateholm in Sweden and Vedbæk in Denmark) and the Baltic area (e.g. Donkalnis in Lithuania), although the amount of burial features, number of grave goods and chronology may vary. Several excavated and analysed contexts with human remains reveal multiple burial practices in Northern Europe (e.g. Brinch Petersen 2015; Gumiński and Bugajska 2016; Tõrv 2016; Butrimas 2016; Ahola 2019). The best known burial sites from the Mesolithic and Neolithic are cemeteries with inhumation burials. These cemeteries share some common features, such as the use of red ochre and the ornamentation of the body with beads and pendants of animal-derived materials.

The variation in the quantity and quality of grave goods has led to the idea that this material, par excellence, can be used for studying social matters. Binford (1971) used the types and amounts of various grave finds to analyse social identity, and this idea has been applied in archaeology in different ways ever since (e.g. O’Shea and Zvelebil 1984; Newell et al. 1990; David 2016). The number of grave goods (mainly animal tooth pendants, bone, antler and stone tools and weapons and osseous anthropomorphic and zoomorphic figurines) has been regarded as an indicator of wealth or social standing in the community at Yuzhniy Oleniy Ostrov (YOO) (O’Shea and Zvelebil 1984). Also, special types of grave goods, like exotic raw materials or pieces of art, have been regarded as indicators of rank in Vlasac and Lepenski Vir in Serbia (Cristiani and Borić 2017; Borić and Cristiani 2019). Furthermore, choices between various raw materials, manufacture techniques, artefact types and costume details have been seen to reflect norms and attitudes relating to identity, origin, kinship and social roles (e.g. Newell et al. 1990; Vanhaeren and d’Errico 2005; Choyke 2013; Mannermaa et al. 2017).

Body adornment is an active means of engaging in social behaviour, and pieces of garments accepted as burial clothes usually have strong symbolic meanings (e.g. Cristiani and Borić 2017). Tooth pendants and their various compositions, raw materials and manufacturing techniques could have provided means for prehistoric hunter-gatherers to express their social identity or origin, based on the levels of individual, family or band (e.g. Mannermaa et al. 2017). By studying wear, we can estimate whether pendants could have been made (and used) by the buried person or whether they were made for the burial. Our microwear analysis of the YOO pendants (Rainio et al. forthcoming) indicates that they were used on a daily basis or in ceremonies before being deposited in burials. The use of tooth pendants on a daily basis has been recently suggested also by Osipowicz et al. (2019) in their study of drilled tooth pendants found in the Šventoji settlement area in Lithuania. Artefact compositions, such as tooth ornaments, could have been part of costumes and headgear used in rituals and ceremonies, and their rattling sound could have played an important role in these activities (Rainio and Mannermaa 2014; Rainio and Tamboer 2018). By studying how tightly pendants were attached and how intensively they were worn, it is possible to estimate how “noisy” the pendants were (Rainio et al. forthcoming).

### Geographical setting

**Fig. 1:**
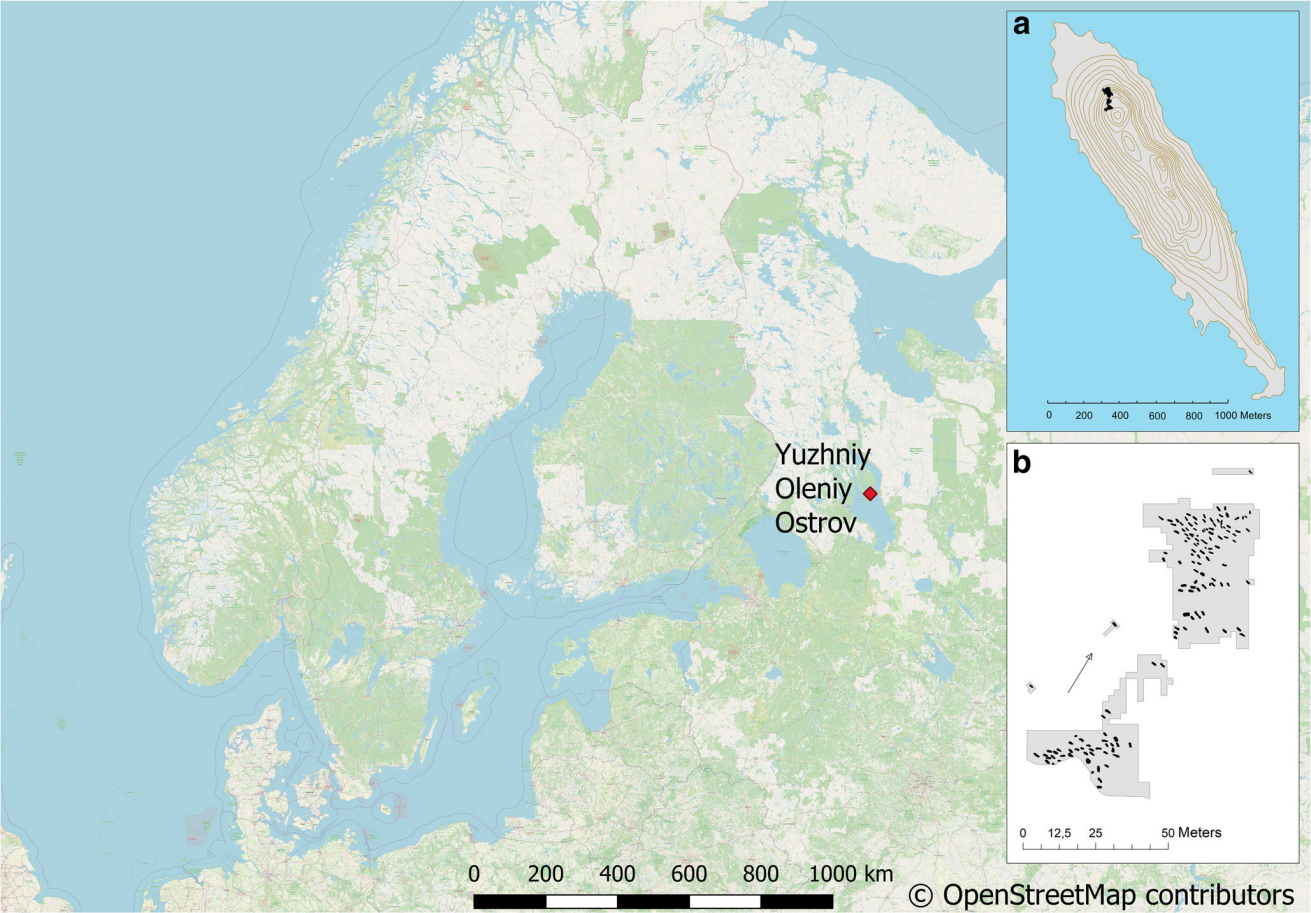
Location of Yuzhniy Oleniy Ostrov in Lake Onega, Northwest Russia, and the distribution of graves (according to Gurina 1956). Map: Johanna Roiha

Yuzhniy Oleniy Ostrov is an island in the north-east corner of Lake Onega in the Karelian Republic, Leningrad region, Northwest Russia (Fig. 1). The geographical coordinates of the cemetery are 62° 07 ′44.3″N and 35°34 ′33.9″E. Today, the island has a length of 2.5 km, a greatest breadth of 0.7 km and a maximum height of 15 m above sea level (Jacobs 1995, p. 362). At c. 6200 cal BC, the island was approximately 1.27 km (length) and 0.27 km (width) in size (Stoliar 2001). The soil sediments of the island are composed of glaciofluvial gravel and sand (Gurina 1956). The island had two hill tops, both a few metres above the water level. The cemetery was located on the northern slope of the highest hill in the north-west part of the island (Ravdonikas 1956) (Fig. 1). The southern part of the burial area was partly destroyed by a limestone quarry pit.

### Cemetery of Yuzhniy Oleniy Ostrov

Yuzhniy Oleniy Ostrov (YOO) is the largest known Late Mesolithic burial ground in Northeast Europe. The area with excavated burials, 177 in total, is approximately 2350 m^2^(Ravdonikas 1956). Altogether, 177 burials of men, women and children were recovered in the archaeological rescue excavations by Ravdonikas (1956) and Gurina (1956) in 1936–1938 (see also Yakimov 1960; Jacobs 1992). Part of the area was already destroyed by sand extraction by local people when the excavations started, and the cemetery was presumably much larger than it appears today (Ravdonikas 1956). According to the analysis by Jacobs (1995, p. 375), approximately 87% of the deceased are adults and 13% are children. Of the adult individuals, 46.5% are estimated as males, 34.7% as females and 18.8% as unidentified (Jacobs 1995, pp. 375–376). Ancient DNA analyses of the YOO humans suggest that the deceased have heterogeneous genetic backgrounds (Der Sarkissian et al. 2013; Semenov and Bulat 2016, p. 43).

Most of the graves at YOO have been furnished with grave goods of stone and osseous materials (Gurina 1956). The majority of the osseous materials are teeth of the Eurasian elk (*Alces alces*), Eurasian beaver (*Castor fiber*) or brown bear (*Ursus arctos*), fashioned into pendants by either grooving or perforating the root tips. The total known number of such tooth pendants there stands at approximately 6000, while one single grave can contain several hundreds of them. Moreover, occasional bones and artefacts made of teeth of wild reindeer (*Rangifer tarandus*), grey wolf (*Canis lupus*), dog (*Canis familiaris*) and wild boar (*Sus scrofa*), as well as bones of several birds, are present in the graves. Also, grooved bone pendants made of irregular long bone splinters, the os hyoideum of ruminants and the ulna of Eurasian beavers are present. Other osseous artefacts found in graves are hunting weapons like barbed and unbarbed points, harpoons and their fragments, as well as anthropomorphic and zoomorphic figurines. The stone artefacts are, for example, finely made flint arrowheads, large flat slate artefacts with holes on one end and quartz utensils. The total number of finds in graves at YOO exceeds 7000, of which more than half are elk teeth. Fish bones or mollusc shells, or artefacts made of such materials, have not been found in burials at YOO.

A new program for studying the chronology of YOO at the University of Oxford, UK, has confirmed a period ca. 100–250 years (c. 6250–6000 cal BC) for the use of the cemetery (Schulting et al. in prep.). The narrow time frame of the burial activities and the high number of graves offer a unique possibility to study kinships and social diversity in a Mesolithic context. In previous publications, it has been suggested that YOO was used by a socially complex hunter-gatherer society (Gurina 1956; Khlobistina 1978; O’Shea and Zvelebil 1984, p. 37; Jacobs 1995). In these studies, the society using YOO has been described as ranked and unequal. Two clusters of burials have been recognized in the cemetery. They have been called the southern group (graves 1–47, 1a, 2a, 3a, 170 “snake clan”) and the northern group (graves 48–165, 167, 76a, 118a, 122a “elk clan”) (Gurina 1956; O’Shea and Zvelebil 1984). In their systematic analysis of find materials, O’Shea and Zvelebil (1984 p. 35) conclude that the population buried in the cemetery may have comprised key actors in the exchange of stone raw materials, namely, slate and flint.

Landscapes are socially constructed phenomena (Zvelebil 1997), and being ritualized landscapes, cemeteries may hold important spatial information of social structures. Cemeteries may have been divided into family- or kin-governed areas. For example, the Khanty in Central Siberia maintain family clusters within communal graveyards, sometimes using fences to separate them (Baltzer 1980 p. 81). Among the Evenk in Eastern Siberia, the cemetery was divided between families, and graves were marked with family signs (Grøn 2015). Transgressing the boundaries of commonly accepted family areas was strictly forbidden. If someone, for example, accidentally buried a relative in their neighbour’s area, or even visited it, it was an accepted practice to punish that person even by killing them (Grøn 2015). At YOO, the graves overlap each other only rarely, indicating that they originally had some kind of markers on the ground, perhaps wooden piles or statues. Obviously, the location of each grave was not accidental. Based on present knowledge, however, it is not known who these people were or if some of them were related to each other. Apart from multiple graves, links between individual burials have not been established.

### The aim of this study

Previous studies have divided the YOO cemetery into southern and northern parts, but so far it has not been possible to associate individual burials with each other. This paper uses elk incisor pendants to identify intra-burial and inter-burial clusters. We study the manufacture technology of the incisor pendants and the amounts and spatial distribution of their technological types relative to the graves. The idea originates from two observations that we made during our use-wear study of a sample of 100 elk incisors from four YOO burials (Rainio et al. forthcoming). First, we observed that one basic technique—namely, grooving—was seemingly used to produce all elk tooth pendants (Mannermaa et al. 2017). Second, we observed that one specific sub-technique of grooving—related to the number, location and depth of the grooves—dominated in each of the studied graves.

Following these two observations, we formulated three hypotheses: (1) Making elk incisor pendants by grooving was a cultural feature of the population or populations using the cemetery. A strict norm prevailed for this basic technique, excluding the use of another possible technique, perforating. Yet, how many grooves were made per tooth and on which side or sides of the tooth root were not strictly controlled. Thus, several sub-techniques as well as a certain degree of freedom existed. (2) The observed variation in the making of the grooves could perhaps indicate discrete subgroups—families, kin groups or such—in the cemetery. Different grooves and groove types could perhaps be part of the cultural communication system, with conventions learnt from the closest relatives or allies and reproduced over and over again more or less consciously. Although comprising small and hardly visible details, the groove types can also indicate specific tying methods of the pendants, attachment systems or ornament sets that visibly characterized groups of people. (3) The decisions regarding the groove types may have been based on functionality, durability and practical solutions.

The aim of this study is to investigate whether the intra-burial and inter-burial distribution of the discovered groove types forms distinguishable clusters or patterns in the cemetery. Close scrutiny of a large amount of tooth pendants, practical experiments and a spatial examination of the findings can reveal whether decisions about groove types were based on functionality and practicalities or personal choice and preferences, as well as other possible regulations, possibly on a kinship level. Creating a typology based on how grooves were made on the elk incisors, we investigate variation in the number, location and depth of the grooves and the distribution of different groove types in individual graves as well as at the whole burial site. Furthermore, we take into account the total number of elk teeth in the graves. We also carry out experiments with modern elk teeth and discuss the functions, meanings and purposes of the tooth pendants found in YOO graves.

## Material and methods

A total of 177 burials have been excavated at YOO. Of these, 144 burials contain artefact finds, while 33 do not have any finds. More specifically, 84 of the burials with finds have elk teeth, of which 77 were available for our analysis. According to Gurina (1956), altogether 4726 elk tooth pendants were excavated at YOO. Our analysis includes 4014 pendants stored at the Peter the Great Museum of Anthropology and Ethnography (MAE), St Petersburg, which were made available for us in 2017–2018. Finds from three burials (numbers 45–47) are deposited at the Karelian National Museum in Petrozavodsk; they were excluded from our study. Gurina’s burial plans reveal the general distribution of the elk tooth pendants in burials, but because the precise location of each individual elk tooth was not documented during the fieldwork, their contexts, position and sequence in the clusters remain unknown. Elks have eight permanent incisors (or six incisors and two incisiform canines), and all types were used for making pendants at YOO.

All 4014 pendants were studied in MAE with the naked eye and an eye loupe with magnification of × 7. The number, location and the approximate depth of the grooves were documented with three scales (< 1 mm, 1–2 mm, > 2 mm). We also documented other traces of processing and wear, such as wear marks on the root tip or surface or narrowing of the root by grinding. A typology was created according to the location and amount of grooves. We ended up using four types related to the location of the grooves on mesial, distal, labial and lingual root surfaces (A, B, C, D, respectively). Combinations of these types were marked with corresponding letter combinations in alphabetical order. The combination ABCD, if forming one continuous groove, was simplified by designating it as type E. In addition, we added five types related to other traces on the root surface: grinded (H), worn root surfaces (I, J), a drilled or carved hole (O) and an unworked root (Y). Descriptions of types are given in Table 1.

**Table 1:**
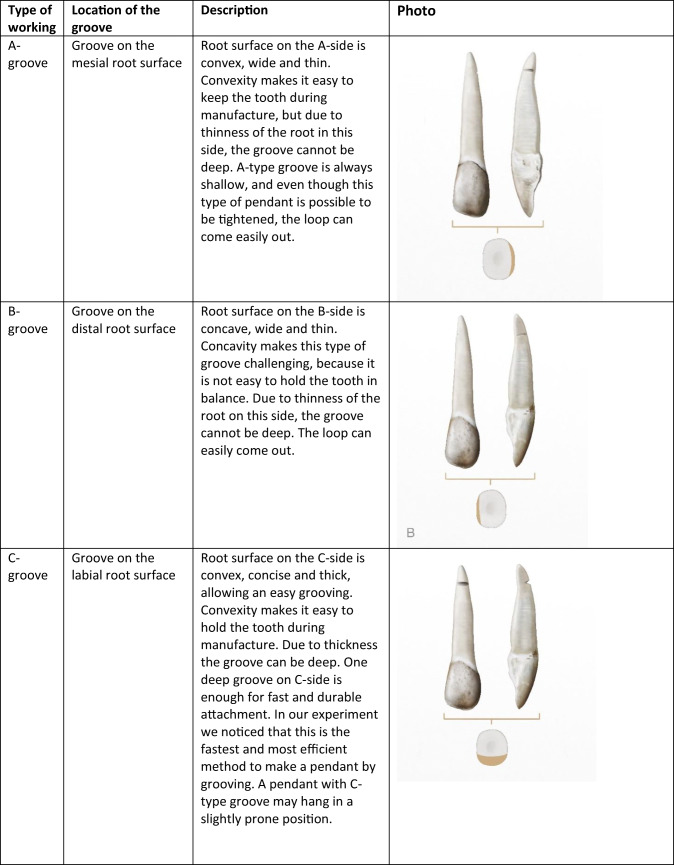

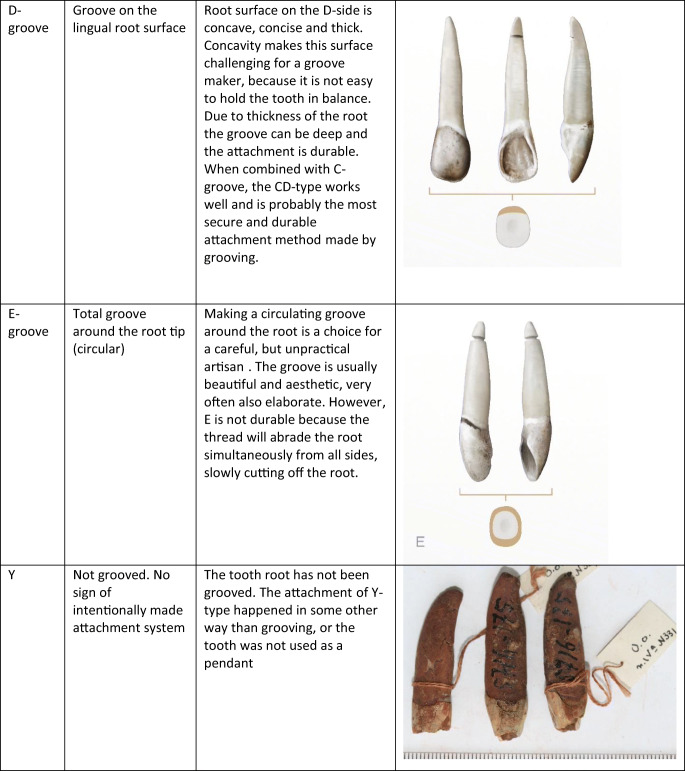

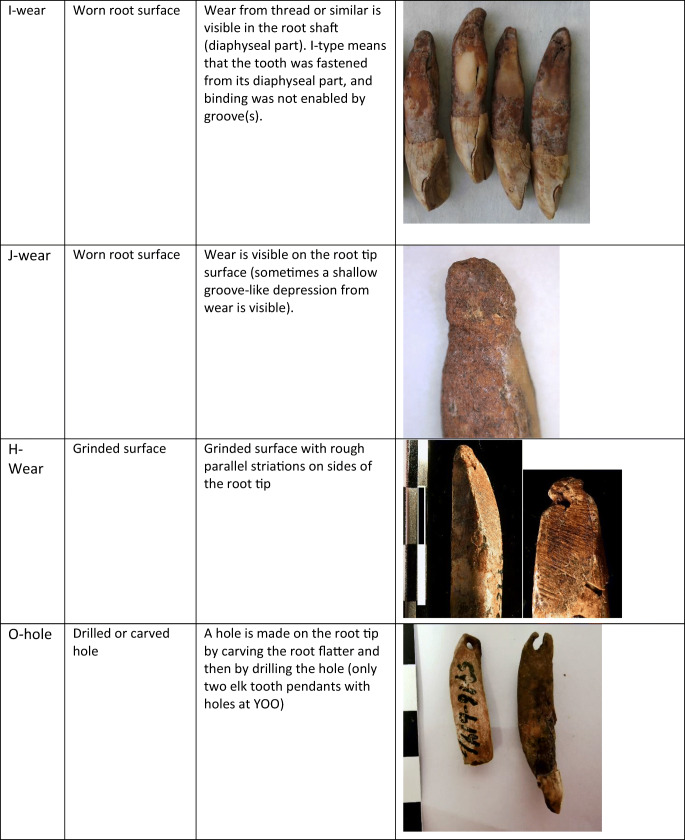
Groove types used in this analysis

Drawings Tom Björklund. Photos by the authors

Although all available teeth in MAE (4014 in total) were scrutinized, 2347 of them appeared to be fragmentary and were excluded from our analysis. Thus, the total number of classified teeth was 1668. Moreover, after analysis, graves with intermingled multiple burials or less than eight classified teeth were considered non-representative due to a considerable share of broken teeth. In five graves, the number of teeth exceeded the number of teeth given by Gurina for this grave (perhaps due to mixing of the finds during storage). All of the graves where materials were obviously mixed or where the majority of the elk tooth pendants were broken were excluded from the final comparison.

A number of graves and teeth in this comparison are 34 and 1352 elk teeth, respectively. By predominant groove type, we mean a type that has at least 50% representation in a grave. The number of elk teeth per grave is adopted from Gurina (1956). The sex and age estimations of humans used in the study are based on the published osteological and genetic data (Gurina 1956; Yakimov 1960; der Sarkissian et al. 2013). The rough age ranges used here are child, young adult, adult, mature adult and senile adult. Derived from the traditional Russian scheme provided by Dr. Vyacheslav Moiseyev (Peter the Great Museum of Anthropology and Ethnography, St. Petersburg, pers. comm. December 2016), the rough limits of the ranges are child (детский I, детский II) 0−14 years, young adult (юношеский ) 15−19 years, adult (возмужалый) 20−35 years, mature (зрелый) 35−55 years and senile (старческий) over 55 years old.

In order to better understand the manufacture and attachment methods of the YOO tooth pendants, we conducted experimental grooving of modern Eurasian elk teeth purchased from licenced hunters in Finland and Russia. A total of 40 incisors from five different mandibles were grooved outdoors using flint blades and modern needle files and following the example of the groove types discovered in the YOO material. The pendants were also wrapped with suspension loops of artificial sinew thread (0.2–1 mm in diameter), twirled around the root tip and knotted tightly. After that, the pendants were suspended from leather patches for wearing experiments (see Rainio et al. forthcoming). Although very little is known about the original attachment methods of the pendants, and there are obviously plenty of options such as tying, lacing, knotting, gluing and crimping, our reasoning in the experiments started from the premise that the grooves were equipped with suspension loops that were tight enough to hold the teeth in place well. All our observations and considerations are based on this basic fact. Tying pendants with threads and strings appears to have been by far the most common attachment method in ethnographical beads, necklaces and chest and garment ornaments (e.g. collections of MAE, Tropenmuseum, National Museum of the American Indian). The experimental grooving and wearing of elk incisors can help to understand the decision and formation processes that went into the marks made on the teeth.

### Experimental grooving and tying

Making a groove with a flint blade (c. 60 × 20 × 1–10 mm) appears to be a fairly simple and straightforward process, taking only a few minutes. The exact time needed depends largely on the number, depth and location chosen for the grooves. The most appealing sides of the elk incisor root for grooving are the mesial and labial sides with convex contours. While grooving these sides, the opposite distal and lingual sides with concave contours help to keep the tooth steady in place. Grooving the distal and lingual sides is possible but not as simple, because the tooth easily slips from the hand. The thin mesial side enables the making of a shallow groove less than 1 mm in depth (type A), whereas the thick labial side allows for a deep groove 1–3 mm in depth (type C), leaving space also for another securing groove on the opposing lingual side (type CD). The thin distal and thick lingual sides similarly allow for shallow or deep grooves (types B, D), but these grooves are, as noted, more difficult to make. Making a circular groove around the whole root tip (type E) is more arduous than the other types and also risky, because it makes the tip very thin and prone to breakage.

All of these groove types can accommodate a suspension loop that holds well. Even a single groove on one side of the root is good enough, provided that the groove is deep and the string in it is knotted tightly. A shallow groove can also work for a while, but in this case, the loop can suddenly come out. The safest way to attach the string is to make a perforation in the root, but that is a more delicate and time-consuming process than grooving. However, this process can be facilitated by narrowing the tooth root by abrading or grinding it into an even surface before starting the drilling (type H). Although little is known about Mesolithic tying methods, our experiments show that the groove type can affect the position of the hanging pendant: while a pendant with a C or CD groove tends to hang at a certain stable angle, a pendant with an A groove tends to hang at right angles. A pendant with a circular E groove, on the other hand, rotates rather freely in several directions.

## Results

### Abundance of groove types and wear traces

**Fig. 2:**
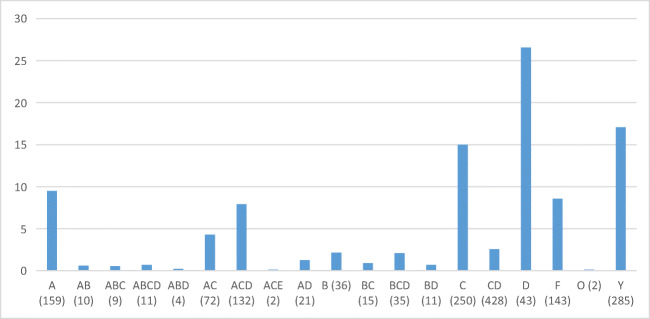
Distribution of groove types on Eurasian elk incisor pendants in graves at Yuzhniy Oleniy Ostrov, in per cents (all elk teeth)

The distribution of 1352 analysed elk teeth according to groove types is shown in Fig. 2. Type CD, composed of two grooves on opposite sides of the root, appears to be the most common type, while type Y, marking teeth without any grooves, is the second numerous. Interestingly, 28% of the type Y teeth have symmetrical wear type J (shallow groove-like depressions) on the root tip or I in the middle part of the root, which probably result from suspension loops and binding. Type C, consisting of a single groove, is also fairly common, followed by types A, ACD and E. These teeth with grooves also show occasional traces of wear from suspension loops and binding. Finally, types AC, B, BCD and D are less numerous, and types AB, ABC, ABCD, ABD, AD, BC and BD are represented by only a few teeth. As a whole, the material appears to show considerable variability.

According to our measurements, most of the types have a characteristic depth of grooves, ranging from shallow A, B and D grooves (< 1–2 mm) to deep C grooves (1–3 mm). Circular E grooves and CD, AC and ACD grooves (1–2 mm) fall in between these extremes. These differences are easily explained by the oval cross section of the elk incisor root, which allows for different depths in accordance with the grooved side (see “Experimental grooving and tying”).

**Fig. 3:**
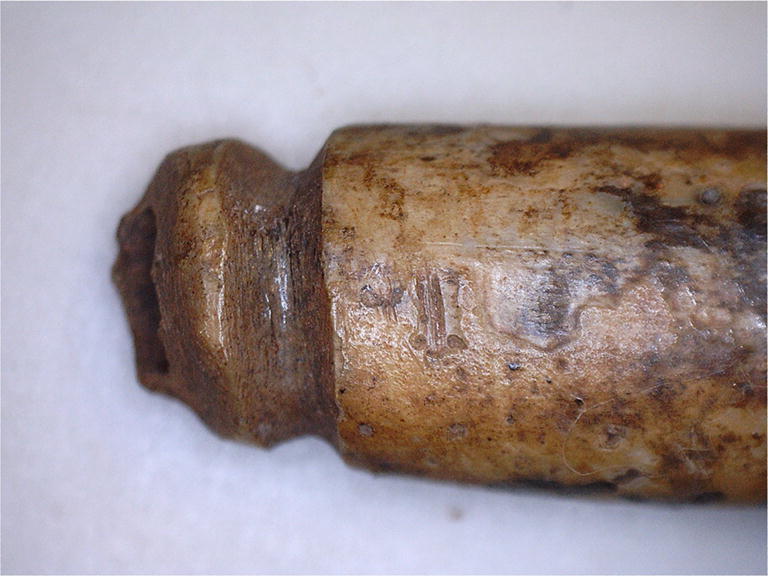
Broken and repaired E groove-type pendant from grave 125 at Yuzhniy Oleniy Ostrov. Photo: Riitta Rainio

The study material contains quite many broken tooth pendants that have been worn through the circular E groove and “repaired” by fashioning similar or other types of grooves after breakage (Fig. 3). These broken pendants are especially common in grave 125, where they comprise 60% of all E pendants. Here, many E pendants also show a special kind of wear in the distal part of the root, just below the crown, comprising a deep semi-spherical furrow that almost cuts off the tooth root. The reason for this wear remains unclear.

Only two elk teeth in the study material, both deciduous incisors, have holes instead of grooves for fastening the pendant. Both of these teeth are derived from burial 127 (Table 1). The holes have clearly been drilled and the root tips narrowed before that by rough abrading or grinding (H). Traces of similar grinding, comprising even, abraded and striated surfaces, are also found in 22 other teeth in the same grave, but these teeth do not have holes. One of these teeth has an unfinished hole on the ground side, while all of them have grooves (C, CD) on the unworked sides. Thus, the grooves do not seem to have any relation to the process of grinding.

### Predominant types in graves and their spatial distribution

The majority of the graves included in the comparative analysis, namely, 20 graves out of 34, have one predominant groove type (Appendix). These burials are numbers 25, 50, 65, 67, 68, 69, 70, 76a, 85, 100, 102, 114, 118, 118a, 119, 125, 134, 151, 156 and 157. The predominant types are A, ACD, C, CD, E and Y, that is, the same as the most common types. Ten of these 20 burials have 75–100% predominance of a particular attachment type (numbers 50, 65, 67, 76a, 102, 134, 114, 119, 125 and 156). The predominant types in these burials (with 75–100% predominance) are again the same: types A, ACD, C, CD, E and Y. For example, burial 65 has a 94% predominance of type C, burial 76a has a 77% predominance of type A, burial 102 has a 95% predominance of type CD, and burial 125 has a 92% predominance of type E. This is remarkable because the total number of classified teeth in these graves is high: 33, 74, 62 and 49, respectively. The average predominance percentage per grave is 58%. The graves with predominant types belong to both adult and mature males and females.

**Fig. 4:**
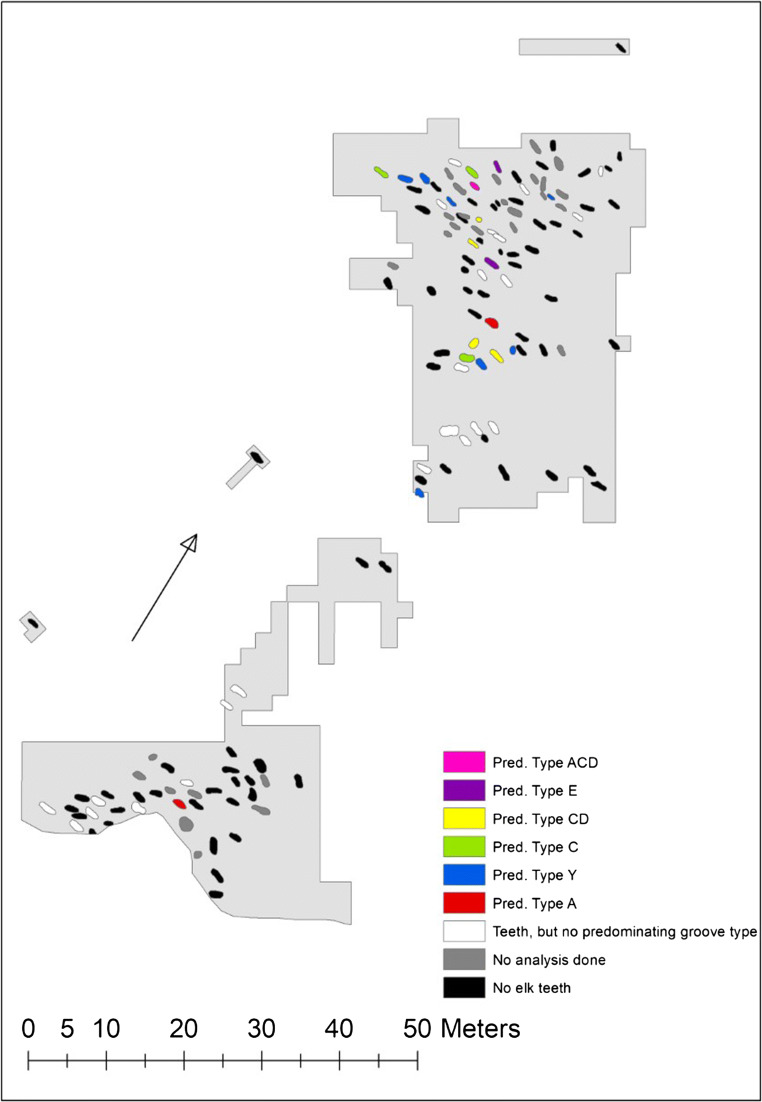
Spatial distribution of predominant groove types on Eurasian elk incisors in graves at Yuzhniy Oleniy Ostrov. Map: Johanna Roiha

Unlike our original hypothesis, the spatial distribution of the graves with predominant types does not show significant spatial clustering (Fig. 4). In four cases, two graves located close to each other have the same predominant type with relatively high predominance percentages: numbers 68 and 69 (type CD with 66% and 62% predominance), numbers 100 and 102 (type CD with 56% and 95% predominance), numbers 118 and 118a (type C with 63% and 69% predominance) and numbers 151 and 156 (type Y with 63% and 75% predominance). In graves 68 and 69 as well as in graves 118 and 118a (located on top of each other), the combination and distribution of all types is almost identical: in the former case CD+C+Y and in the latter case C+CD+ACD/BCD. Another noteworthy pattern is that all graves with predominant types, except for two, are located in the northern part of the cemetery or, more precisely, in its centre (graves 65, 67, 68, 69, 70, 76a, 85, 100, 102, 114, 118, 118a, 119, 125, 134, 151, 156 and 157). In the southern and marginal parts of the cemetery, graves 25 and 50 with predominant types have the predominance percentages of 54% (type A) and 100% (type Y). The other graves in these areas have predominance percentages (like 42%, 44%, 26%, 41%, 39%, 22%, 24% and 32%) and show a fairly wide range of types per grave. For example, grave 9 shows types A, C, CD, D and E, and grave 61 shows types ABD, ACD, C, CD and E, with all these in almost equal proportions. This type of heterogeneity is rare in the northern part of the cemetery, especially in its centre. The average predominance percentage in this central area is 66% (graves 64, 65, 67, 68, 69, 70, 76, 76a, 85, 97, 100, 102, 107, 114, 118, 118a, 119, 125, 127, 134, 151, 156 and 157), while in the rest of the graves, it is 41% (graves 1a, 4, 9, 14, 25, 47, 50, 58, 59, 61 and 147). The average predominance percentages in the northern and southern parts, as established in earlier research, are 61% and 41%, respectively.

### Age, sex and amount of pendants in graves

**Fig. 5:**
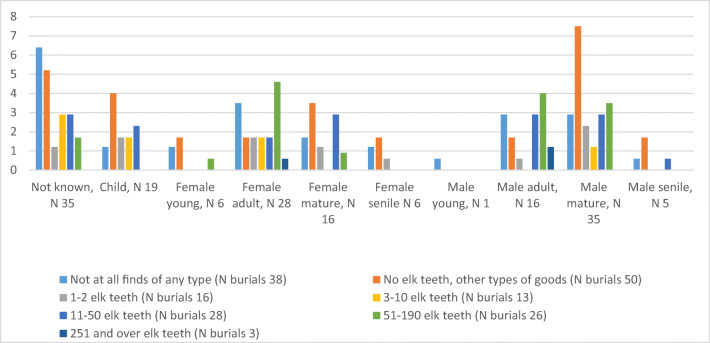
Number of Eurasian elk teeth in graves at Yuzhniy Oleniy Ostrov, based on age and sex of the deceased (in per cents). Age and sex according to Gurina 1956, Yakimov 1960

**Fig. 6:**
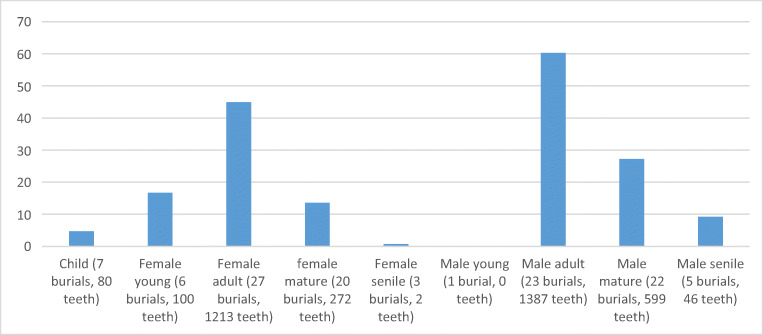
Average sum of Eurasian elk teeth in graves at Yuzhniy Oleniy Ostrov, based on age and sex of the deceased (in per cents)

Elk teeth are most abundant in the burials of adult females and adult and mature males (Fig. 5). A very high number of elk teeth, around 300 specimens, are present in three burials: grave 107 contains an adult female, and graves 85 and 100 contain adult males. None of the children have a high number of elk teeth in their graves. Burials without any finds are present in all sex and age groups. Burials without elk teeth but with other find types are mainly the case among children and mature females and males (especially the males). One or two elk teeth are found in the graves of all age and sex groups. The average sum of elk teeth in burials reflects their relative abundancy in various sex and age groups (Fig. 6). The highest average sum of elk teeth is found in adult male burials (60.3 teeth) and adult female burials (44.9 teeth), followed by mature male burials (27.2 teeth) and young female burials (16.7 teeth). A very old person rarely has teeth in their grave: the average for senile males is 9.2 and senile females 0.7.

The average number of elk teeth in child burials is 4.7. These numbers indicate a clear pattern: pendants seem to have been in possession of persons who died at a virile age. Interestingly, the only identified young adult male in the cemetery does not have any elk teeth. However, despite these statistics, we can conclude that the number of elk teeth separates individuals rather than age groups. For example, some mature and adult males’ graves have no elk teeth at all, and some have many. It also has to be noted that the age and sex groups are not equally represented, and this could affect the results.

**Fig. 7:**
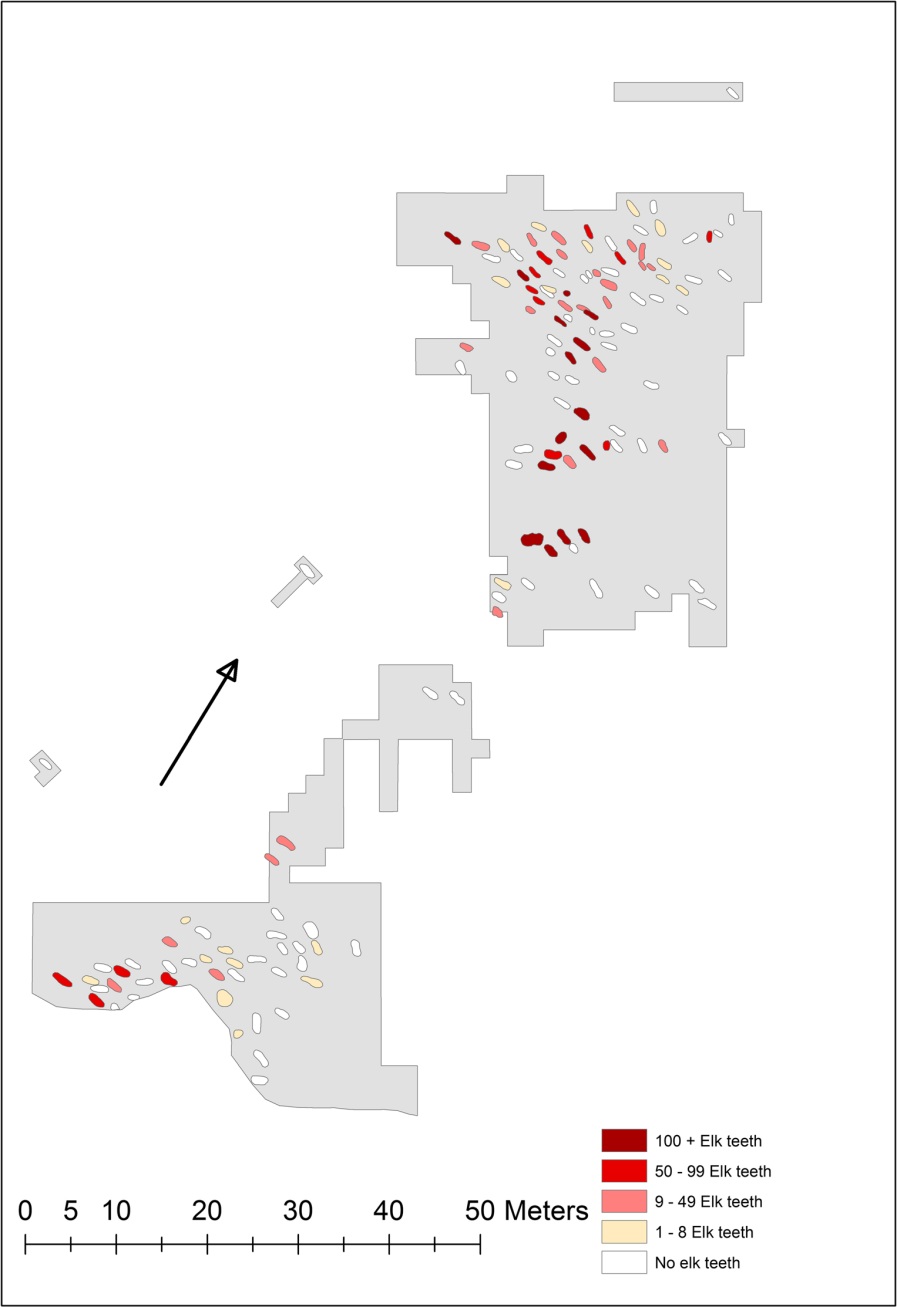
Number of Eurasian elk teeth in graves at Yuzhniy Oleniy Ostrov. Data and map according to Gurina 1956. Map: Johanna Roiha

In addition, a clear pattern is that the graves in the northern part of the cemetery contain more elk teeth than the graves in the southern part (Fig. 7). The average number of teeth per grave is 31 in the north and 10 in the south. Furthermore, the graves with the greatest number of teeth, more than 120 specimens, are situated in the centre of the northern part, that is, in the same area as the graves with predominant types. In many cases (numbers 68, 69, 76a, 85 and 100), the graves are actually the same.

## Discussion

### Is pendant technology a suitable method to study social differences in a mortuary context?

In contrast with the results we obtained from our preliminary analysis (Rainio et al. forthcoming), our systematic analysis of all elk teeth does not indicate that grooving techniques were indicative of an intra-site division of distinct groups (families or kin). If the groove technology of the elk teeth was family or clan based, this is not evident in our results. It is also possible that groove technology was family or kin based, but the deceased at YOO were not related to each other by family or social ties.

While groove analysis is laborious, it can significantly help in studying how teeth were turned to pendants and how they were used or whether they were used. When interpreting the results of our analysis and estimating the relevance of the method used, we have to pay attention to the quality of the material. Several aspects affect the results. First, the fractured state of many pendants diminished the sample size. Many pendants had to be excluded from the analysis because their roots were broken. Second, the fact that not all individuals have been aged and sexed means that several burials had to be excluded from the analysis. Age and sex groups are not equally represented at YOO, which also can affect the results. Finally, the excavated burials represent only a sample of the original burials in the area. Despite the high number of burials and investigated pendants, the material still represents only a share of the original material. Our data might look quite different if all pendants had been included in the analysis and especially if the whole material from the original cemetery area would have been available.

Taking the limiting factors mentioned above into consideration, we suggest that the attachment manufacture techniques of the tooth pendants can still be indicative of distinct groups in the cemetery, and our method can be applied to other sites as well. Manufacture technique analysis of elk tooth pendants can provide important data about their uses and meanings, as well as about the humans who manufactured or used them.

Earlier studies have raised question whether the humans buried at YOO only represent certain members of the groups living in the Onega area. These members may have represented the noble part of the population, like shamans (Gurina 1956) or tradesmen (O’Shea and Zvelebil 1984) or the family members of these groups. It is also possible that the population buried at YOO represents clans and families in the larger area without any special emphasis on the noble part of the community. This latter interpretation is in line with the cultural homogeneity of the deceased, emphasized by Jacobs (1992 p. 397), but it is partly in contrast with the idea of cultural diversity suggested by O’Shea and Zvelebil (1984).

While aDNA results suggest multi-ethnic origins for the people buried at YOO (der Sarkissian et al 2013), the elk tooth pendants indicate a homogenous material culture. Perhaps a common material culture was a way to moderate genetic variation and maintain an experience of unity? While grooves in the pendants comprise only small visual details, how they are made reflects a routine and perhaps a learned, experiential and possibly unconscious tradition. This reflects strong cultural homogeneity, a situation that may be explained by the short use time of the cemetery. These results are not necessarily mutually exclusive. Hunter-gatherers were very mobile (e.g. Pitulko et al. 2019), and the intensive network of waterways connecting Lake Onega across a huge geographical area in all directions offered easy routes for people to move, build contacts and mix genes with each other.

### Tooth pendant technology as cultural communication

Earlier research has indicated that the tooth pendants found in graves are often heavily worn (Larsson 2006; Rainio and Mannermaa 2014; Rainio et al. forthcoming). This indicates that the studied pendants were not made specifically for the funeral but used earlier in life, perhaps being sewn to clothing, either by the deceased person before death or by fellow members of the community. As indicated above, we suggest that the pendants had important functions during the lives of the individuals who were buried with them at YOO. The precise meanings and functions remain unknown, but they may be associated with cultural communication and identity. The groove type would have been hidden under the attachment substance (knotting, lacing, gluing or crimping) and probably not visible at all. However, the position of the groove (groove type) affects the position of pendants when attached to necklaces or clothing. In this way, the appearance of the elk tooth ornamentation and its rattling may have been signs associated with cultural communication and expressions of social identities (potentially on the level of kin or rank). Clothes can be signs of clan or family or kin, and technology and production were important paths to physical engagements with the environment and the world (Newell et al. 1990; Dobres 2000; Jensen and Grønnow 2017). The uniformity of the chosen species (elk), the tooth type (incisor) and the technique (grooving) indicates that strict norms prevailed in the industry of tooth pendants at YOO. This speaks for high cultural homogeneity of the group or groups that used the cemetery.

The controlled use of manufacture technology for producing elk tooth pendants suggests that these pendants, and how they were made and used, were an expression of the identity and origin of the buried person (on the level of the individual, family or band). Such a strict control is not reflected in the manufacture of brown bear canines; their technology is multiple and contains various types of grooving, perforating and leaving the root unworked (Gurina 1956, pp. 134–138). The notion that strict control was important in making elk tooth pendants is important. It accords well with the proposed central role of elk in the economies and social and religious cultures of north-eastern European forest areas (Taavitsainen 1980; Ukkonen and Mannermaa 2017; Seitsonen et al. 2017). The central role of elk in rituals is supported by the predominance of elk figures and depictions over any other animals in northern mobile art (e.g. Mantere and Kashina 2020) and in Fennoscandian, western Russian and central and eastern Eurasian hunter-gatherer rock art (e.g. Okladnikov 1970 pp. 89–104; Helskog 2012 pp. 218–229).

### Number of tooth pendants associated with lived life?

On the basis of our results, we can conclude that the elk teeth are associated with lived life. The number of elk teeth in graves seems to vary between individuals, and not between age groups. For example, some mature and adult males have no elk teeth at all, and some have many. The individuals with the highest number of elk teeth are males and females at a virile age. Also, some children have elk tooth pendants in their graves, indicating that at least some members of these populations started to gather pendants already at a young age. However, none of the child graves have a high number of pendants, perhaps because children did not have time to collect them.

Interestingly, very old age did not increase the amount of elk teeth. If we accept the idea that pendants were personal belongings and that the amount of such belongings was dependent on personal achievements, we can suggest that during their life, these persons were not in a position that allowed them to receive (a high amount of) elk tooth pendants.

### Were groove types related to practicality, durability and the artisan’s personality*?*

As demonstrated in our experimental study, practical reasons related to manufacture technology can affect the chosen groove type. Factors like time and the energy invested in making the pendant required durability of the attachment system, while the desired position of the hanging pendant could have affected the choice of how the groove was made or if the groove was made at all. In the following, we survey the groove types discovered at YOO from this practical perspective, trying to understand their distributions and functions.

Types CD, Y and C are the most common groove types at YOO, followed by types A, ACD and E. In the case of types C and CD, this is understandable, because these grooves are easy to make, deep and enable a tight and safe fastening. Wear mark J, (traces of suspension loops on the ungrooved sides of 8% of the C pendants) proves that this attachment system held well in reality. In the case of type Y, the prevalence is not as readily understandable. While the unworked Y teeth did not require much preparation, they often have wear mark J or I around the root tips or in the middle part of the root, probably resulting from suspension loops and binding. This also indicates that these teeth were threaded as pendants and carried on clothes, ornaments or accessories, obviously for quite a long time, because such abrasions do not develop quickly. How the grooveless Y teeth could have been threaded tightly remains an open question. Perhaps gluing or some other technique was used to secure the suspension loops. In previous studies, elk or other animal teeth without grooves or holes have not been interpreted as pendants at all (e.g. Jonuks and Rannamäe 2018).

As a common and even predominant type, the A groove is similarly surprising, because it is impossible to make a deep and safe groove on the mesial side of the elk incisor root. This apparent problem was not insurmountable for the people of YOO, however, as evidenced by the Y pendants above. They had some tying or tying-and-gluing solution that remains inexplicable. On the other hand, our tying experiments indicate that the A pendant tends to hang differently from C and CD pendants, namely, on its side. Thus, it seems possible that the A groove was made when the aim was to keep the pendant in a side position, for example, as some type of special ornament. Alternatively, there may have been no need for a durable attachment, or the groove maker was just careless. Types ACD and AC can be regarded as more careful versions of type A. In these cases, the A groove was made more secure by adding C or CD grooves on both sides of the A groove.

Type E means that a circular groove was carved around the whole root tip, carefully and patiently, so as to not break the thin root walls. The artisan must have been cautious and hard working but also unconcerned about practical aspects, because the groove was easily worn through this type of pendant. Why were so many E grooves made nevertheless? The E groove has a symmetrical and harmonious appearance. In practice, however, the grooves were almost invisible, because of the suspension loops. Hanging pendants of the E type rotate freely, probably producing a stronger sound than other types. In the YOO material, we see several cases in which pendants with E grooves have been broken and remodified by making similar or simpler grooves. This supports our earlier observation that most of the pendants at YOO were used before deposition in the grave (Rainio et al. forthcoming). The repairing of functional artefacts indicates their importance and intensive and/or long-lasting use. Repaired pendants were perhaps owned by an old person, or they circulated from one person to another. For example, burial 125 has many pendants with traces of both E and other types of grooves. Apparently, these pendants were prepared carefully for a special ornament and used heavily by several persons and/or during a long period. Burial 125 is one of the four “vertical” burials at YOO (Gurina 1956); in these burials, the deceased was probably buried in a half-sitting position. The skeleton in burial 125 belonged to an elderly man (Gurina 1956, Fig. 64). Many special features of the grave—a sitting position, a high number of elk teeth (83), as well as the carefully designed and perhaps inherited elk tooth ornamentation—together indicate that this man was a very important person.

The B groove, alone or combined with other grooves, is significantly less abundant. The rarity of this type is not surprising, because it is inconvenient to make a groove on the concave distal side of the elk incisor root. In addition, the groove on this side is shallow and insecure in use. In fact, we think that the occasional B grooves might have even been accidental or made chiefly in incisors with exceptionally straight roots.

What does it mean that all but two elk incisors at YOO were grooved and not perforated? To make grooves instead of holes could have been done to turn a tooth into a pendant quickly. If one wants to produce a high number of pendants in a short time, grooves seem to be a good choice. Then, how should we interpret the presence of two elk tooth pendants with drilled holes in grave 127? Both of these teeth with holes are deciduous incisors. They are smaller than permanent incisors, and the root walls are thinner. To make holes in deciduous elk incisors without breaking the root tip is even more demanding than to make holes in permanent teeth. We can assume that the person who drilled holes in these elk teeth was experienced in drilling, perhaps an outsider or a newcomer. Although grave 127 stands alone by having two perforated elk teeth, making holes was not exceptional at YOO. Many of the massive bear canines found in this cemetery were perforated, and their root tips were narrowed before this by rough abrading or grinding (H). Interestingly, traces of similar grinding can be found on a total of 24 elk incisors in grave 127. Two of them are perforated, one shows an unfinished hole on the ground side, and the rest have grooves on the unworked sides. This creates an impression that all of these teeth were perhaps meant to be perforated, but for some reason, the maker changed the plan. Was drilling elk teeth too difficult? Was drilling elk teeth inappropriate after all? The deceased in grave 127 is an adult woman. She has 90 elk teeth along her thighs and between her knees probably forming the remains of a decorative fringe on an apron (Gurina 1956, Appendix, p. 362, Fig. 65, Yakimov 1960). Moreover, a few elk teeth lie on her right humerus. Three grooved bear tooth plates were found on the left lower femur. Bone pieces were found on the chest and red ochre covered the skeleton.

Thus, the groove types preferred by the Mesolithic makers can be at least partially explained by practical reasons, such as the convenience, quickness, firmness and durability of the attachment. However, sometimes the logic of the makers remains inexplicable to us, probably due to our limited understanding of the tying, lacing and sewing methods or the deeply rooted cultural traditions of the time. In some cases, the chosen groove types appear to give clues on the personal characteristics or personality of the artisans, based on how much attention was devoted to finalizing minute and hardly visible details like grooves. We even catch a glimpse of the decisive moment, when the artisan wavered between the two choices: whether to drill or to groove.

### Groove type predominance related to the composition and number of tooth ornaments?

When observing the elk incisor pendants in their archaeological find contexts, the most distinct pattern is that one of the common groove types—either CD, Y, C, A, ACD or E—tends to dominate per grave. On average, the proportion of the predominant type is 58%, but in many graves, with dozens of analysed teeth, the proportion is as much as 75–100%. The graves with the highest values are mostly located in the northern part of the cemetery or its centre. What do these predominance values mean? The most logical explanation is that the incisor pendants found in the same grave were grooved more or less on one occasion, possibly by one person and/or during a relatively short time. Indeed, sometimes the grooves in a grave are so similar that they seem to result from serial production. Because a large number of the pendants at YOO show traces of wear (with I and J resulting from suspension loops and binding), this production was hardly related to burial preparations or the making of burial clothes. Instead, it could be connected with the making of particular garments and the tooth ornaments attached to them. To validate this idea, we have to survey possible garment remains in the YOO graves.

**Fig. 8:**
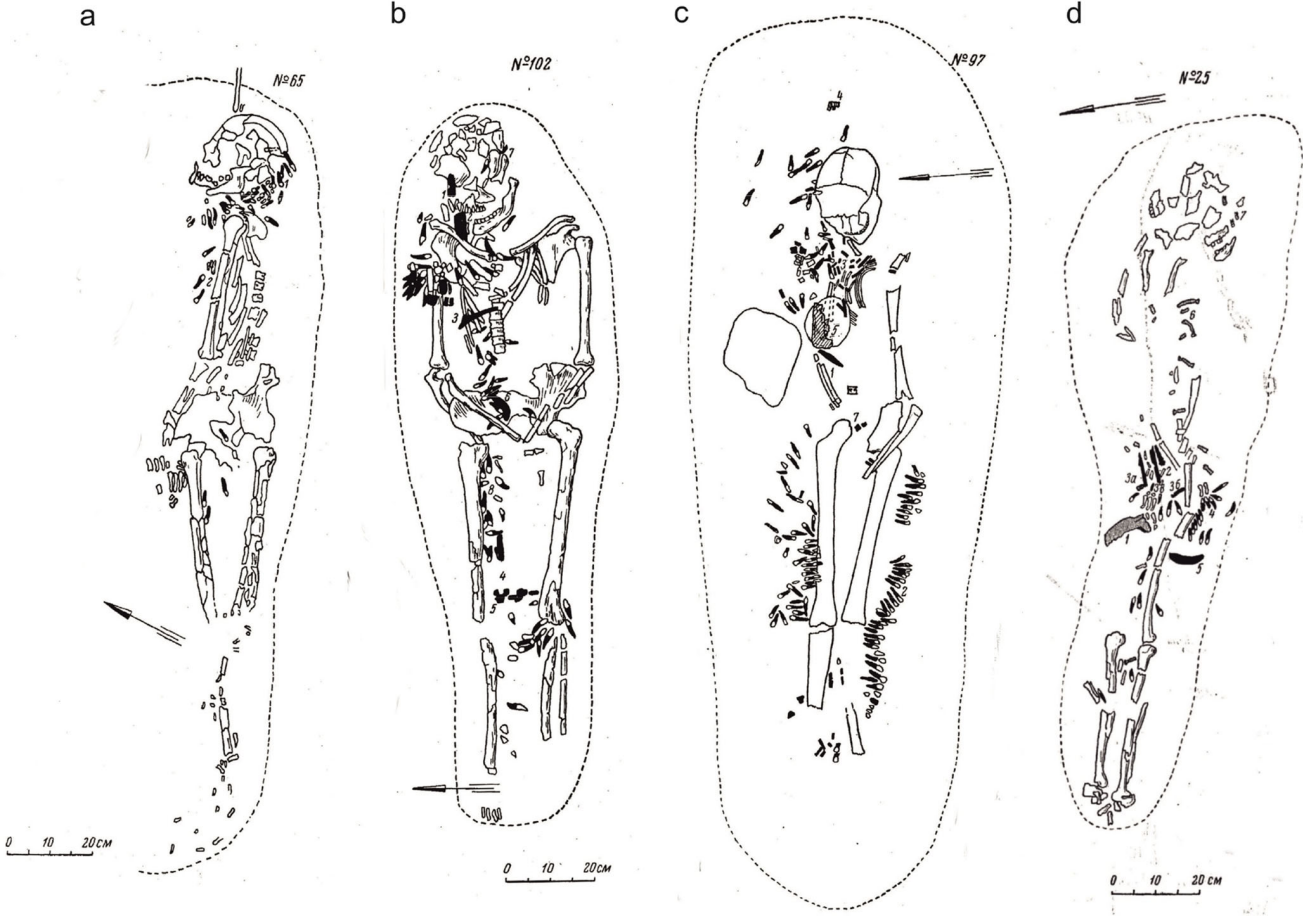
Drawings of graves 65, 102, 97 and 25 at Yuzhniy Oleniy Ostrov. Adapted from Gurina 1956, Fig. 32, 51, 23a and 13

In 24 of our 34 analysed graves, it is possible to have a rough idea of the position of the tooth ornaments in relation to the overall costume or body parts of the deceased. In graves 65, 67 and 156, almost all the tooth pendants are clustered on the neck of the deceased, forming remains of some type of necklace or collar (Fig. 8a) (Gurina 1956, Fig. 32, 33, 75). The predominance percentages in these graves are 94%, 84% and 75% and the predominant groove types C, Y and Y, respectively. In graves 50, 76a and 102, the majority of the tooth pendants are clustered on either the right or left side of the body from the neck to the thighs, creating the impression of a long fringe of a robe or blanket (Fig. 8b) (Gurina 1956, Fig. 25, 39, 51). The predominance percentages in these graves are 100%, 77% and 95% and the predominant types Y, A and CD. Moreover, graves 1a, 97, 107, 127 and 147 appear to have a couple detached clusters of tooth pendants, as it were reflecting separate tooth ornaments on different body parts (Fig. 8c) (Gurina 1956, Fig. 1, 23a, 53, 65, 74). Interestingly, the predominance percentages in these graves are remarkably lower (only 42%, 46%, 38%, 29% and 24%). This decrease is explained by the fact that the pendants in these graves fall into two or three major groove categories. All this suggests that the tooth ornaments at YOO were composed of teeth with similar grooves and made at one time, almost routinely, by one person. Thus, the tendency of one groove type to dominate is mainly associated with a single, homogenous tooth ornament in that grave, whereas lower predominance values are associated with several ornaments per grave. Individual random groove types in graves may represent later additions to replace broken or separated teeth. On the basis of these find contexts, it also seems evident that the groove types as such had no connection with particular ornaments, garments or hanging positions. A necklace, for example, could be composed of either C or Y pendants and an apron or a hip ornament of either A, ACD, C or Y pendants (Fig. 8d) (Gurina 1956, Fig. 13, 57, 59, 61). Consequently, the Y pendants in adjacent graves 151 and 156 appear as two completely different ornaments by the skeletons, namely, a necklace and some type of hip ornament.

The making of large numbers of composite tooth ornaments at once means that a bulk quantity of elk incisors was available for the artisans at the same time. As one elk has eight permanent incisors (or six incisors and two canines), the ornaments in graves 65, 76a and 102, for example, required the teeth of no less than 8–18 individual elks. This implies systematic storage or extremely successful hunting seasons, the outcome of which is revealed in spectacular ornaments on costumes and accessories and eventually in graves. However, a few graves with lower predominance values diverge from this overall picture in that they appear to contain a single tooth ornament with a variety of groove types (Gurina 1956, Fig. 6, 8, 13). These graves (numbers 9, 14 and 25) are mostly situated in the southern part of the cemetery or in the margins of the northern part. How to explain them? Following the same logic, it might be assumed that these ornaments were made during less successful times or emergency periods, by attaching teeth one by one or alternatively by people who did not have access to all the assets of the community. This assumption might get some support from the fact that the graves in the southern and marginal areas, in general, have less elk incisors than the graves in the central area. Many of the graves in the marginal areas lack any elk teeth. Thus, there appears to be a positive correlation between the number of elk teeth per grave and predominance percentage of groove type per grave. This spatial pattern resembles, although it is not identical to, the earlier division of the cemetery into distinct southern and northern groups or clans (O’Shea and Zvelebil 1984).

## Conclusions

YOO is an ideal site for systematic analyses of tooth pendants because of the high number of burials made during a short chronological time span and the high number of elk tooth pendants deposited over a relatively short period. Unfortunately, many of the elk tooth pendants from the YOO burials are broken and could not be used in our analysis. An important finding of our analysis is that groove types did not reveal clusters which could be associated with kinship. In a couple cases, two closely situated graves have the same predominant groove type. An interesting pattern is that most burials with one predominant type are located in the so-called northern group. This supports previous observations suggesting a centre or “hotspot” in the northern part.

Another important finding is that grooving is really the only method of making elk tooth pendants seen at YOO, with only two exceptions. This indicates a degree of shared aesthetics of the population and that norms regulated pendant manufacture. Such unity is also indicated by the restricted use of almost exclusively specific teeth from elks, beavers and brown bears for making tooth pendants. The third finding is that the most common groove type is the most efficient and handy one to produce and at the same time the most secure and durable. This indicates that pendant production was routinized and that pendants were an important part of the social identity of the people using YOO. This also supports that pendants were intended to last for a long time, perhaps one individual’s lifetime or even from generation to generation.

Based on our observations, we suggest that elk teeth were associated with the lived life of the buried people and that pendants were personal belongings of the deceased. The amount of elk teeth clearly divides the deceased. Because the amount of teeth in the graves does not increase with the age of the deceased, the elk teeth cannot be understood only as signs of accumulated wealth or prestige gained during life. Their importance was something more profound and meaningful than a mere symbol of wealth. For example, some children have elk teeth, and some children do not have elk teeth. On the other hand, the graves of adult and mature men may contain plenty, few or occasional teeth, or they may completely lack elk teeth. Despite the number of elk teeth varying between burials at YOO (with some graves having no elk teeth at all), we do not think that this variation alone can be used as evidence of the social identity or “social rank” of the person. In our opinion, all individuals buried at YOO were somehow special and selected (maybe based on their special social roles, or their family or kin bonds). For some still unknown reason, these persons were chosen to be buried on this cemetery island, unlike the majority of their community members.

In sum, our detailed analysis supports the results from earlier studies that ornamentation and personal adornment formed an integral part of social identity and personhood. Animal tooth pendants can be sorted into types, based on their abundancy, distribution and manufacture technique. Such types can help to identify patterns (e.g. clan-based or family-based divisions). In our materials, even the teeth without any grooves show wear, indicating that they were also attached to clothes or accessories.
